# Long-term monitoring of the Iberian ibex population in the Sierra Nevada of the southeast Iberian Peninsula

**DOI:** 10.1038/s41597-020-0544-1

**Published:** 2020-06-25

**Authors:** José Enrique Granados, Andrea Ros-Candeira, Antonio Jesús Pérez-Luque, Ricardo Moreno-Llorca, Francisco Javier Cano-Manuel, Paulino Fandos, Ramón C. Soriguer, José Espinosa Cerrato, Jesús María Pérez Jiménez, Blanca Ramos, Regino Zamora

**Affiliations:** 1 Parque Nacional y Parque Natural de Sierra Nevada. Ctra. Antigua de Sierra Nevada Km 7, 18191 Pinos Genil, Granada Spain; 2 Wildlife Ecology & Health group (WE&H), Jaén, Spain; 3 Laboratorio de Ecología (iEcolab), Instituto Interuniversitario de Investigación del Sistema Tierra en Andalucía (CEAMA), Universidad de Granada, Avenida del Mediterráneo s/n, 18006 Granada, Spain; 4 Grupo de Ecología Terrestre, Departamento de Ecología, Universidad de Granada, Facultad de Ciencias, Campus Fuentenueva s/n, 18071 Granada, Spain; 5 Agencia de Medio Ambiente y Agua de Andalucía. Servicios Centrales, C/Johan G. Gutenberg 1, Isla de la Cartuja, 41092 Sevilla, Spain; 6 Estación Biológica de Doñana (CSIC). Avenida Américo Vespucio s/n, 41092 Sevilla, Spain; 7 Departamento de Biología Animal, Biología Vegetal y Ecología. Universidad de Jaén. Campus las Lagunillas, 23071 Jaén, Spain

**Keywords:** Conservation biology, Population dynamics

## Abstract

This dataset provides long-term information on the presence of the Iberian ibex (*Capra pyrenaica hispanica* Schimper, 1848) in Sierra Nevada (SE Iberian Peninsula). Data on the abundance and demographic structure of the Iberian ibex population were compiled over the last three decades. Transects were laid out to record different variables such as the number of individuals sighted, the perpendicular distance of each group of Iberian ibex to the transect line and sex as well as age of individuals in the case of males. These data enabled the calculation of population parameters such as density, sex ratio, birth rate, and age structure. These parameters are key for Iberian ibex conservation and management, given that Sierra Nevada harbours the largest population of this species in the Iberian Peninsula. The data set we present is structured using the Darwin Core biological standard, which contains 3,091 events (582 transect walk events and 2,509 group sighting events), 5,396 occurrences, and 2,502 measurements. The occurrences include the sightings of 11,436 individuals (grouped by sex and age) from 1993 to 2018 in a total of 88 transects distributed along Sierra Nevada, of which 33 have been continuously sampled since 2008.

## Abstract (summary)

**Table:** 

Measurement(s)	population • biological sex • age • distance
Technology Type(s)	visual observation method
Factor Type(s)	year
Sample Characteristic - Organism	Capra pyrenaica
Sample Characteristic - Environment	mountain range
Sample Characteristic - Location	Sierra Nevada

Machine-accessible metadata file describing the reported data: 10.6084/m9.figshare.12310463

## Background and Summary

The Iberian ibex, one of the bovine species of the genus *Capra* in Europe^1^, is an endemic ungulate of the Iberian Peninsula, distributed in different mountain ranges^2,3^. Sierra Nevada harbours the most numerous population of Iberian ibex, with the greatest genetic variability of the Iberian Peninsula^4,5^. This population has increased in the last 40 years from a density estimated in 1960 of 1.29 ind/km^2^ to some 11.68 ind/km^2^ estimated in 2012. Considering the results found during the demographic monitoring, the Iberian ibex population of Sierra Nevada can be considered somewhat stable with a slight increase over the last 20 years^2,3^. The population trends of this species in Sierra Nevada in recent decades appear to be related to land-use changes and human depopulation^2,3^.

This dataset provides historical and recent information compiled over the last few decades on the population abundance and demographic structure of the Iberian ibex population of Sierra Nevada, this being the largest population reservoir of the species. This is the most complete time series of those that we have found available for a wild ungulate population. This continuous monitoring has enabled us to analyse and estimate population trends, relating demographic information to changes in land-use transformations and/or climate. This information is essential for the management of this Iberian ibex, an iconic animal species for conservation as well as hunting. Long-term research and monitoring are essential for understanding the dynamics of populations, communities, and ecosystems^6,7^. Additionally, the use of information from long-term data series enables managers to evaluate and mitigate threats to ecosystem function and services while operating more effectively in the legal and political arenas^8^. In Sierra Nevada, we have launched a long-term monitoring programme to provide a more encompassing evaluation of ecosystem function and services under a global-change context^9,10^.

**Fig. 1:**
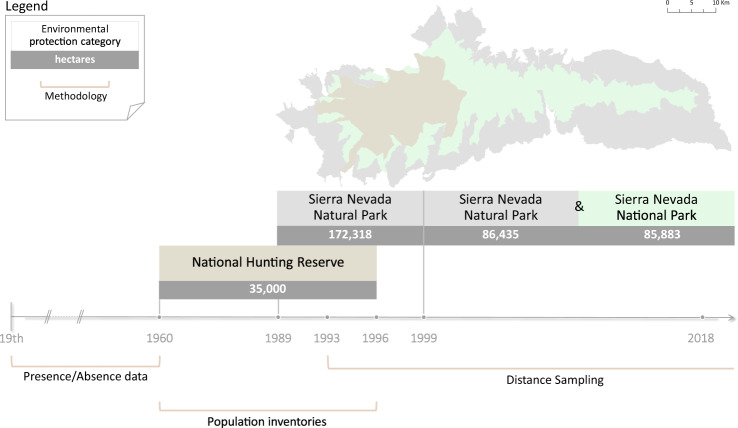
Timeline of the different stages in the monitoring of the *Iberian ibex* population in Sierra Nevada, illustrating changes in environmental-protection categories and in the methodology used to estimate the number of individuals.

Four different stages in the monitoring of the Iberian ibex population can be considered chronologically in Sierra Nevada (Fig. 1), characterized by changes in environmental-protection categories and the methodology used to estimate the number of individuals. An initial stage (19th century to first half of the 20th century) includes Iberian ibex presence or absence data in particular sites of Sierra Nevada. The second stage (1960-1996) comprises the population inventories of the number of Iberian ibex present in the 35,000 hectares of the former National Hunting Reserve. The third stage began in 1989 with the declaration of Sierra Nevada as a Natural Park, with an area of ca. 172,000 hectares, where Distance Sampling has been undertaken since 1993 to estimate the size of this Iberian ibex population. The fourth stage began in 1999 with the declaration of part of Sierra Nevada mountain range as a National Park. This new category of protection has not entailed changes in the methodology used to estimate population size, although it has implied a new management model combining one territory where sport hunting is prohibited (National Park) with another where this activity is permitted (Natural Park). This sector has been favoured by improved management of the species as a whole.

The presence of the Iberian ibex in Sierra Nevada has been known since the early nineteenth century^11–16^. Schimper^17^ described the subspecies *Capra pyrenaica hispanica* in 1848 from specimens captured in Sierra Nevada. In 1885, Sánchez García^18^, in a brief review of the mammals and birds that frequent the province of Granada, described the presence of this species from the north-western part of Sierra Nevada to south-eastern area of Almería province, including all the mountain ranges on the coast. Other references^19–29^ describe the presence of the Iberian ibex in the Penibaetic Mountain System.

However, the most accurate scientific studies of this ungulate population in Sierra Nevada started in the middle of the 20th century. In his book on the bouquetin of the Alps (*Capra ibex*), Couturier^30^ dedicated a chapter to the Iberian ibex, studying several specimens from Sierra Nevada. According to this author, the population consisted of about 600 individuals, although higher densities had previously been reached. From the beginning of the 1960s, when Sierra Nevada was declared a National Hunting Reserve^31^, the Iberian ibex population was being intensely monitored, combining the epidemiological vigilance with ecological, demographic, and reproductive aspects.

**Fig. 2:**
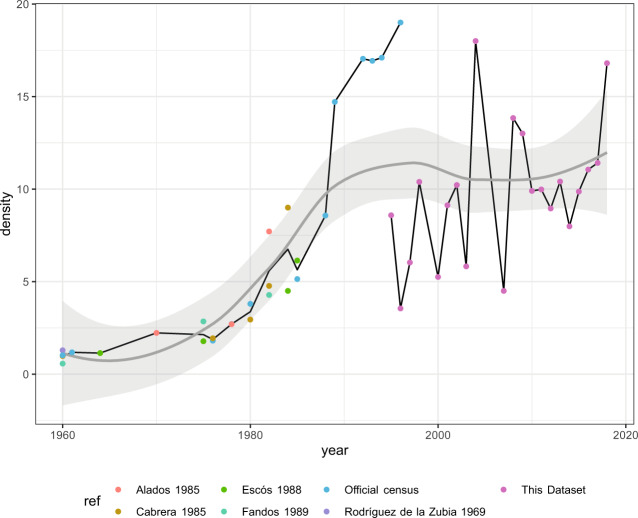
Time course of the *Iberian ibex* population in Sierra Nevada since 1960 combining density values from the literature and estimates from the dataset we present here (pink dots). Points show density values (ind/km^2^). Different colours indicate the bibliographic source. There is a discrepancy between official census and the density values of this dataset because they are based on different methodologies and also due to the different areas covered (see Fig. 1). The trends of the Iberian ibex population in Sierra Nevada appear to be related to land-use changes and human depopulation.

Several authors^32–40^ have studied the size and characteristics of this Iberian ibex population. Although some of these authors have focused on specific areas or refer to the whole population of this species in the Iberian Peninsula. The densities estimated for the population from Sierra Nevada in the studies outlined above are shown in Fig. 2, together with the densities calculated from the dataset we describe here (1993–2018).

From 1993, the methodology for estimating the size of the Iberian ibex population in this mountainous area changed. For this, a sampling based on the implementation of linear transects was designed, collecting field data on: the number of individuals observed, sex, age in the case of males, the perpendicular distance of each group of Iberian ibex to the transect line, and other relevant observations such as the presence of lesions caused by sarcoptidosis.

Population density was calculated, using specific programs such as TRANSECT or DISTANCE in different versions. The rest of the parameters that define the population, i.e. sex ratio, birth rate, and age pyramid (for males), were estimated as indicated in the Usage Notes section. The global analysis of these parameters was decisive in new management guidelines, adapting them to theoretical values. This management approach is useful in handling the increased probability of the appearance and transmission of infectious diseases, while avoiding the habitat deterioration.

These field data have been used totally or partially in some publications, most of these being technical documents^41–46^.

The dataset described here contains annual observations for each transect. In total, 11,436 individuals of Iberian ibex were recorded since 1993 in a total of 88 transects. The Open Access publication of these historical data constitutes a valuable resource for consultation by scientists and managers, guaranteeing its conservation through free and open access (without legal or economic restrictions) to the different sectors of society. The data can be reused to generate new information useful for the management of these Iberian ibex in Sierra Nevada and similar mountains.

## Methods

### Study area

**Fig. 3:**
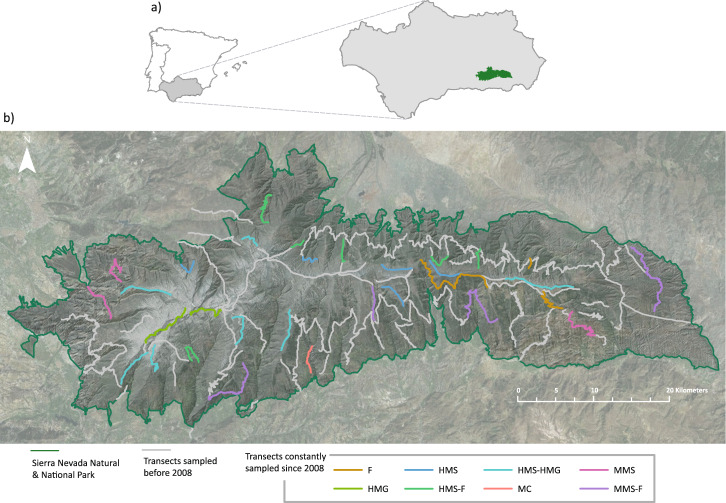
(**a**) Location of Sierra Nevada and (**b**) distribution of the 88 transects sampled, highlighting the 33 transects that have been constantly sampled since 2008, classified by the most predominant habitats in the area covered by each transect. Type of habitats are: F (forest), HMG (high-mountain grasslands), HMS (high-mountain shrubland); HMS-F (high-mountain shrubland and forest), HMS-HMG (high-mountain shrubland and high-mountain grasslands), MC (mountain crops), MMS (mid-mountain scrubland) and MMS-F (mid-mountain scrubland and forest). A more detailed description of these classes can be found in the Habitat description section.

Sierra Nevada is a massif located in the south-eastern Iberian Peninsula (37°14′-36°54′N; 2°37′-3°39′W) within the Baetic System (part of the Penibaetic mountain ranges), near the Mediterranean Sea (Fig. 3a). Sierra Nevada has the highest summits of the Iberian Peninsula, the peak Mulhacén reaching 3,479 m a.s.l., making this the second-highest mountain range in mainland Europe, after the Alps. Though the general climate is Mediterranean, the mountain morphology gives it the characteristics of a continental climate. Biogeographically, five of the six thermotypes defined for the Mediterranean region appear in Sierra Nevada, from the thermomediterranean in the lowest and driest areas of the east to the cryoromediterranean in the highest peaks^47^. The mean precipitation gives rise to a dry and subhumid ombrotype, although exceptions appear due to extreme drought (eastern part) and to areas with mean precipitation of more than 1,000 mm/year. Topographically, the area is heterogeneous, with strong climatic contrasts between the sunny, dry south-facing slopes and the shaded, wetter north-facing slopes.

A major diversity hotspot in the Mediterranean region, Sierra Nevada has unique ecosystems with many endemic species. Overall, Sierra Nevada comprises 27 habitats types from the habitat directive (Annex I of Directive 92/43/CEE). The Iberian ibex is distributed throughout Sierra Nevada from the summits to the bottom of the valleys, depending on the seasonality of the ecosystem.

The presence of Iberian ibex has been constant in this mountainous region, for many reasons, such as: the implementation in the mid-1960s of a set of legislative measures that promoted the conservation of the species, human depopulation, habitat transformation, increase of shrublands, and massive reforestation projects. Regarding the absence of large predators in Sierra Nevada, although the wolf (*Canis lupus*) was abundant during the Middle Ages until nearly the end of the 19th century, hunting caused its disappearance in 1933.

### Sampling protocol

Sampling based on linear transects has been used since the early 1930s. This method has proved practical, efficient, and relatively inexpensive^48,49^. In addition, it has been recommended because it provides the ability to control the reliability of the results^49^, this being the main reason for using the Distance Sampling in the census of wild ungulates^39,50^. In Sierra Nevada, the linear transects method for estimating the size of the Iberian ibex population was first used in 1993^51^. A more detailed explanation of the methodology can be found in the *Plan específico de gestión de la población de cabra montés en el Parque Nacional de Sierra Nevada*^43^ (Specific Management Plan for the Iberian ibex population in Sierra Nevada National Park) and in the monitoring methodologies of the Sierra Nevada Global-Change Observatory^52^.

The transects are sampled by two or more observers, on foot or by vehicle, when terrain conditions allow, at a speed of no more than 15 km/h. The sampling time is adapted to the dates when the field work is carried out, recording the official time in the surveys. In summer, the observers walk the transects mainly at dawn and dusk. In autumn, the sampling time is extended throughout the day. Each transect is sampled on a single day, so the sampling of all of them is completed on consecutive days during about 2 weeks. The optical materials used are binoculars (8 × 35) and a telescope (20 × 40). When circumstances prevent a satisfactory viewing (individuals far away or hidden by the surrounding vegetation, temporary brevity of contact, etc.) sightings are not taken into account.

The sampling design (length and location of transects) was carried out following criteria of randomness and stratification. Although a total of 88 transects were sampled throughout the monitoring since 1993, not all of them were sampled every year: before 2008 due to methodological changes and after 2008 due only to weather conditions. This means that the transects designs were made uniform as of 2008, when 33 transects were fixed and were the only ones constantly sampled from 2008 onwards. Some of these were modifications of the old transects (Fig. 3b), while others had been sampled before 2008 also. Based on our experience, these 33 transects were considered suitable because they cover a large part of the mountain range and they can be sampled in a reasonable time without the resulting in observers fatigue (less than four hours).

Overall, one sampling was conducted annually, although in 1995 two samplings were undertaken (summer and autumn) but none in 1994, 1999, 2005, or 2006. The first years the surveys were conducted in summer so that snowfall would not prevent sampling. From 2008 onwards, the 33 fixed transects are sampled annually in autumn whenever the weather conditions allow. Transects are sampled in autumn (before the oestrous cycle), when animals are more active and more easily observed, offering greater probabilities of detecting animals.

In each sampling, the observers walk the linear transects taking notes on the Iberian ibex groups sighted and recording different variables such as: the number of individuals observed (group size); the contact hour; and perpendicular distance of each group of Iberian ibex to the transect line. At the individual level, records are made of physical condition (mainly the presence of lesions caused by sarcoptidosis), the sex of each Iberian ibex, and the age in the case of the males. In addition, the date as well as the starting and ending times of the sampling are also recorded, as well as the identity of the observers.

The probability of detecting an individual is related to spatial distribution of the sightings^53^ and visibility conditions, habitat coverage, land topography, animal and group size, as well as the density. The method assumes that, if the density is high, many individuals will be sighted up close. If the density is low, only a few individuals will be sighted, and far away. The following premises must be assumed: animals on the transect line are always observed; animals must be immobile when they are observed or located on the spot before they move; no animal should be counted twice; distances and sighting angles must be calculated accurately and sightings are independent events.

With all these data collected, the parameters that define the population were: density (number of individuals/km²), sex ratio (number of females/number of males), birth rate (number of kids/number of adult females) and age pyramid for males. The size of the horns and body morphology make it easier to determine the years of age or age class to which males belong, as in Alados and Escós^37,54^.

### Habitat description

**Fig. 4:**
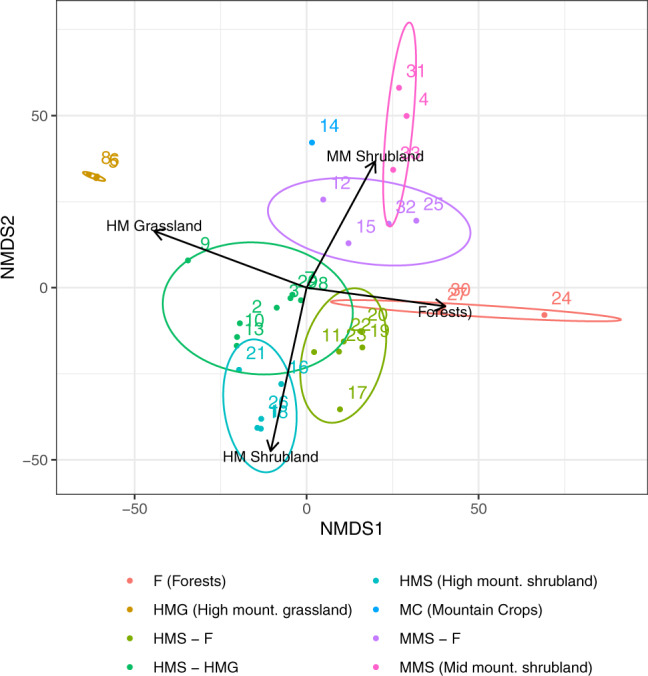
Ordination of the transects (number) according to the classification made. Each colour represents the class with the ecosystems represented.

The 33 transects sampled in the last 10 years (since 2008) cover different habitats and elevational ranges. To explore how the transect covers the ecosystems found in Sierra Nevada, we classified each transect according to the ecosystems it covers. For each transect, we analysed the percentage of each ecosystem sampled. A buffer of 200 m along each transect was calculated. Using the map of the ecosystems of the Sierra Nevada^55^, we calculated the percentage of each ecosystem sampled in each transect. The transects were then classified based on the percentage represented of each ecosystem and the elevational range covered. Finally, a Multidimensional Non-Metric Scaling, using *vegan* R package^56^, was performed to explore and validate the classification made (Fig. 4).

The classes are:

- HMS (High-Mountain Shrubland). Transects situated in 1950-2600m a.s.l. with a predominant habitat of >70% of juniper-genista thickets (high-mountain shrubland).
- F (Forest). Transects with the predominant ecosystem is natural forest (holm oak, *Quercus ilex*; melojo oak, *Quercus pyrenaica*) or pine plantation (*Pinus* sp.).
- C (Croplands). Transects covering mainly mountain crops (53%) with a significant presence of aquatic systems (15%) and mid-mountain shrubland (13%).
- HMG (High-Mountain Grasslands). High-mountain grasslands predominate (>70%) in the transects located at the highest elevations (2650/2800-3300 m a.s.l).
- MMS (Mid-Mountain Shrubland). Transects with a diverse habitat composition but with mid-mountain shrubland as the dominant ecosystem. High abundance of mid-mountain shrubland generally with different forests: pine plantations; autochthonous Scots pine; holm oaks (*Quercus ilex*); and, occasionally with mid-mountain grasslands.

In addition, there are three classes of transition between the different classes, *i.e*. HMS-HMG (High-Mountain Shrubland - High-Mountain Grasslands); HMS-F (High-Mountain Shrubland - Forests) and MMS-F (Mid-Mountain Shrubland - Forests).

### Data management and standardisation

**Fig. 5:**
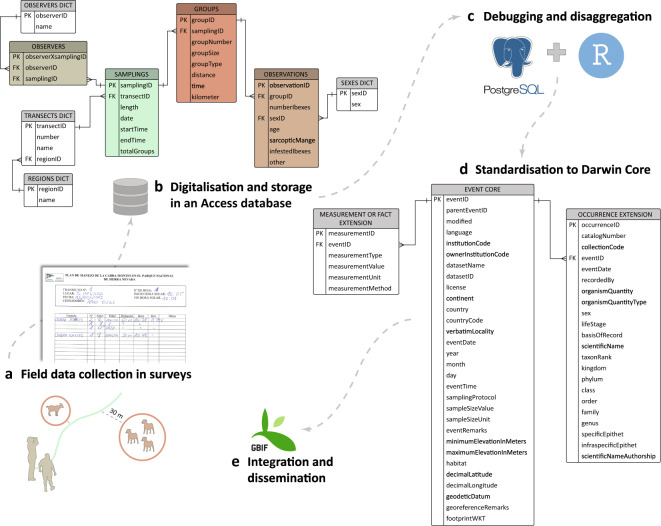
Stages of this life-cycle dataset: from data collection to integration in GBIF.

The publication of historical data of Iberian ibex in Sierra Nevada stored in old formats has the advantage that it not only makes them public, but it also protects them from possible accidental loss^57^. In this case, all field data collected was recorded annually in surveys on paper for each transect sampled (Fig. 5a). To facilitate the digitalisation, a database was designed to store the information generated, using a form. Its structure was designed according to the main elements in samplings and their relations: the observers, the transects, the observed groups of Iberian ibex, and the sightings of the species at an individual level (Fig. 5b).

After data debugging (see Technical Validation section) and disaggregation (Fig. 5c), the dataset was standardised to the Darwin Core^58^ structure as sampling event data (Fig. 5d). The resulting dataset was published through the Integrated Publishing Toolkit^59^ (IPT v2.3.6) of the Spanish node of the Global Biodiversity Information Facility (GBIF) (http://ipt.gbif.es) (Fig. 5e).

This collaborative framework to retrieve and to make the data available to the scientific community responds to the Sierra Nevada Global-Change Observatory efforts to manage data following the FAIR principles^60^. In this case, we seek to enhance the potential reusability of these data by making the dataset:

- Findable: by the integration and dissemination of data and metadata through GBIF with a unique and persistent identifier assigned. The dataset is also hosted by the Sierra Nevada Global-Change Observatory.
- Accessible: by open and free access to the data and metadata.
- Interoperable: by standardising data to the Darwin Core standard and metadata to the EML standard.
- Reusable: by providing a complete provenance and description of the dataset in GBIF metadata sections and in the data descriptor presented here. In addition, in the Usage Notes section, we present a reproducible example of use that begins with the download of the Darwin Core file available in GBIF and shows how to explore some population parameters.

## Data Records

The data descriptor we present corresponds to version 1.7 of the dataset called *Dataset of Iberian ibex population in Sierra Nevada (Spain)*^61^, which can be downloaded as a Darwin Core Archive (DwC-A) through GBIF: 10.15470/3ucqfm (the repository website can be changed to English in the top right corner of the website). This dataset was made available to the public domain under a Creative Commons Zero waiver (CC0 1.0) and it will be updated annually depending on the financial support. The custodian of all the information collected is the Administrative Center of the Sierra Nevada National and Natural Park, whereas the owner is the Department of Agriculture, Livestock, Fisheries, and Sustainable Development.

The DwC-A contains sampling event data, specifically: 3,091 records of events (582 transect walk events and 2,509 group sighting events), 5,396 records of occurrences, and 2,502 records of associated measurements. The DwC-A structure is based on a sampling event hierarchy: the “parent events” are the transect walk events, meaning that two or more observers sample one transect; whereas the “child events” are the group sighting events; that is, an Iberian ibex group is sighted and the observers collect different variables. The occurrences compile the sightings of 11,436 Iberian ibex (grouped by sex and life stage) from 1993 to 2018 in 88 transects distributed along Sierra Nevada. One variable was included in the Measurement or Fact table: the contact distance, meaning the perpendicular distance between each group of Iberian ibex and the transect line.

Clarifications and remarks concerning some Darwin Core elements included in the dataset (Fig. 5d) are provided hereafter:

- The *parentEventID* element makes it possible to create the hierarchy among the transect walk (parent) events and the group sighting (child) events. This element is used to link the different group sightings to the same transect walk where they were recorded. A child event “inherits” the information from its parent event.
- Individuals (species occurrences) with the same *eventID* belongs to the same group (event).
- The *verbatimLocality* contains local reference names, locating the start and endpoint of the transect (e.g. a mountain refuge, a crag, etc.) or a representative place where the transect is located.
- The *minimumElevationInMeters* and *maximumElevationInMeters* elements correspond to the minimum and maximum altitude values of the transect derived from a 10-m resolution Digital Elevation Model (DEM).
- The *habitat* element contains the percentages of the most predominant habitats in the area covered by each transect and the class to which it belongs. This information is available for the 33 transects that have been constantly sampled since 2008. In total, 8 classes were identified after applying a Non-Multidimensional Scaling technique (see Habitat description section).
- The *decimalLatitude* and *decimalLongitude* correspond to the centroid coordinates of the transect (inside), but the complete representation of the shape (multilinestring) can be found in the *footprintWKT* element.
- The local time zone is indicated as an offset from UTC (conforming to ISO 8601) in the time-related elements: *modified*, *eventDate* and *eventTime*.
- The *lifeStage* element was completed in the case of Iberian ibex kids and males, indicating years of age for the latter.

## Technical Validation

Different validation processes were applied in the data cycle stages described in Fig. 5:

- During the sampling, the observers fundamentally cross-checked the sightings *in situ*.
- In the second step, due to the large volume of data, we implemented some controls and validation rules in the Access form in order to reduce human errors and facilitate the digitalisation:

- Input masks control-data entry formats (especially date/time data type).
- We defined required fields (e.g. transect number and sampling date).
- We made lists of predefined values (e.g. group types: male alone, female alone, males, females, females with kids, and mixed groups).
- We established some “control fields”, i.e. variables that the person digitalising the data calculated manually to facilitate the information identification. For instance, before introducing the sightings, the person had to indicate the total number of groups identified in each survey; the size of each group; the type of group categorized by sex and age; etc.

As for transects, a more accurate digitalisation was carried out at a scale of 1:1000 in ArcGIS 10.2^62^, using as cartographic base the orthophotos from PNOA (Spanish National Program for Aerial Orthophoto).

(c) The data were processed through the PostgreSQL relational database management system (RDBMS) version 11.3^63^ together with R version 3.6.0^64^ using the package Rpostgres^65^ and the spatial extension PostGIS version 2.5.2^66^, in addition to other packages: DBI^67^, knitr^68^, dplyr^69^ and splitstackshape^70^. In this way, we created a validation process in R and SQL code to check specific errors derived from digitalisation and corrected them. When necessary, the surveys were re-checked and several validation rounds were run. Specific examples are given below:

- We checked whether all the information was associated: samplings without any observers assigned, groups that had no sightings assigned, etc.
- Regarding null values, we checked whether all the essential variables were filled out, e.g. males without the age variable, groups without the size value, etc.
- We identified any duplicated information.
- We revised any incongruous data, e.g. the hour when a group was sighted had to be between the start and end time of the sampling.
- We also checked the “control fields” because they were susceptible to error, e.g. the automatic sum of individuals might not match the indicated group size; groups categorized as mixed should be males and females with kids, etc.

(d) The Darwin Core was standardised according to the practices recommended by the TDWG guidelines (https://dwc.tdwg.org/terms/).

## Usage Notes

We provided a reproducible example using the data stored at GBIF. The first step was to download the Darwin Core Archive (.zip file) of the dataset from the IPT. Then, using the *finch* package^71^ we processed the Darwin Core Archive (DwC-A) and load the datasets. In the following steps we computed the population structure over the study period and explored several population parameters, such as sex ratio and birth rate.

### Population structure

We explored the time course of population structure. For this, each year, we computed the percentage of individuals belonging to a certain age class. Also, we computed the average of each age class for the study period.

- First, from the *Occurrence* table (from the DwC-A), we selected the field *lifeStage* which indicates the age of the individual.
- For individuals belonging to “kid” *lifeStage*, we considered half to be males, since in many mammalian populations a balance between the sexes is maintained with a 1:1 ratio, which does not differ significantly from a theoretical distribution^50,72^.
- Then, we computed the number of individuals by year and age class, and the percentage.

**Fig. 6:**
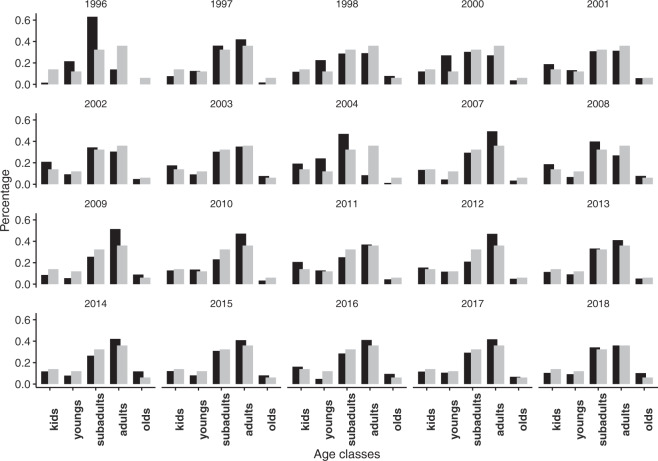
Annual population structure (age classes) of Iberian ibex males on Sierra Nevada. Black bars indicate individual frequency for each age class: kids (0), young (1-2), subadults (3-4), adults (5-8) and old individuals (>8). Grey bars indicate the average frequency for each age class during the period 1995-2018. For visualization, we averaged the age-class frequency over the period of the data set.

Then, we plotted the structure of the population for each year (Fig. 6).

### Sex ratio and birth rate

To compute the sex ratio and the birth rate, we first needed to determine male and female counts grouped by year. We also computed the number of kids per year, using the field *lifeStage* included in the *Occurrence* table.

- Extract year from *eventDate* field.
- Group data by year and determine the male and female counts.

**Fig. 7:**
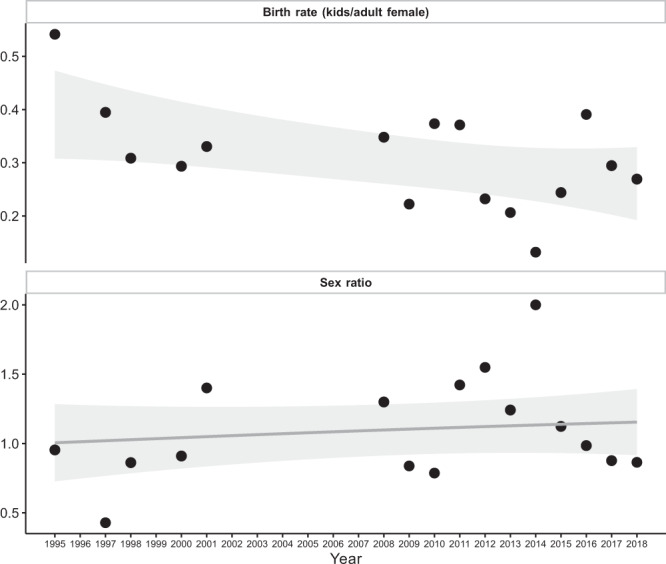
Time course of the birth rate (number of kids/adult female) (top) and sex ratio (female count/male count) (bottom) of the Iberian ibex populations at Sierra Nevada. A GAM smooth was added to improve the visualization.

Then we computed the *sex ratio* (*sr*) as female count/male count and the *birth rate* (*br*) as the kid count/female count. We used the variables *eventDate*, *sex*, *organismQuantity* and *lifeStage* from *Occurrence* table (from the DwC-A). The results are plotted as in Fig. 7.

## Data Availability

The code used in the Usage Notes section is publicly available through the Zenodo repository^73^. The Usage Notes section was performed using R computing language^64^ and the packages: *finch*^71^, *tidyverse*^74^, *knitr*^68^ and *here*^75^.
